# MAVS signaling of long-lived brain-resident myeloid cells is needed during viral encephalitis to adjust the transcriptome of CNS infiltrating CD8^+^ T cells

**DOI:** 10.1186/s12974-025-03497-1

**Published:** 2025-07-07

**Authors:** Andreas Pavlou, Luca Ghita, Felix Mulenge, Inken Waltl, Olivia Luise Gern, Pia-Katharina Larsen, Bibiana Costa, Veronica Duran, Lena Mareike Busker, Shelly J. Robertson, Yvonne Lueder, Stephan Halle, Reinhold Förster, Sonja M. Best, Martin Stangel, Ulrich Kalinke

**Affiliations:** 1 Institute for Experimental Infection Research, TWINCORE, Centre for Experimental and Clinical Infection Research, a joint venture between the Helmholtz Centre for Infection Research and the Hannover Medical School, Hannover, 30625 Germany; 2 Department of Neurology, Clinical Neuroimmunology and Neurochemistry, Hannover Medical School, Hannover, 30625 Germany; 3 Center for Systems Neuroscience, University of Veterinary Medicine Hannover, Hannover, 30559 Germany; 4 Genentech, South San Francisco, CA 94080 USA; 5 Innate Immunity & Pathogenesis Section, Laboratory of Neurological Infections and Immunity, Rocky Mountain Laboratories, National Institutes of Allergy and Infectious Diseases, National Institutes of Health, Hamilton, MT USA; 6 Institute of Immunology, Hannover Medical School, Hannover, 30625 Germany; 7 Institute of Clinical Chemistry and Laboratory Medicine, Hannover Medical School, Hannover, 30625 Germany; 8 Current Address: Translational Medicine, Biomedical Research, Novartis Pharma AG, Basel, 4056 Switzerland; 9 Cluster of Excellence - Resolving Infection Susceptibility (RESIST, EXC 2155), Hannover Medical School, Carl-Neuberg-Straße 1, Hannover, 30625 Germany

**Keywords:** Microglia, CD8^+^ T cells, Brain infection, Cross-presentation, CNS, MAVS signaling, RLR signaling

## Abstract

Neurotropic viruses like vesicular stomatitis virus (VSV) can infect the central nervous system (CNS) through the olfactory route. Following intranasal instillation, VSV moves along the axons of olfactory sensory neurons to the olfactory bulb. While within the olfactory bulb the spread of the virus is controlled by microglia activation and the recruitment of peripheral leukocytes, some of the underlying mechanisms remain unknown. To investigate these mechanisms, we used mice with conditional deletions of the mitochondrial antiviral-signaling protein (MAVS), an adaptor for RIG-I-like receptor (RLR) signaling. By selectively deleting MAVS in neurons, astrocytes, or long-lived myeloid cells, we discovered that RLR signaling specifically within brain-resident myeloid cells is crucial for protection against the virus. Infected mice with a MAVS deletion in these myeloid cells showed normal myeloid cell and leukocyte infiltration into the brain. However, the P2RY12^+^ microglia showed aberrant expression of genes involved in antigen cross-presentation. Furthermore, flow cytometry experiments revealed diminished MHC class I expression on MAVS deficient microglia. Moreover, CNS infiltrating CD8^+^ T cells had dysfunctional transcriptional profiles. Therefore, our findings indicate that during viral CNS infection, MAVS signaling in brain-resident myeloid cells, presumably microglia, is essential for antigen cross-presentation and the relicensing of protective, infiltrating CD8^+^ T cells.

**Supplementary Information:**

The online version contains supplementary material available at 10.1186/s12974-025-03497-1.

## Introduction

Viral infection of the central nervous system (CNS), e.g., with rabies virus, orthoflaviviruses, as well as many other viruses, can result in life-threatening inflammation [1–3]. Currently, the therapeutic arsenal for the treatment of neurotropic virus infection is very limited and many affected individuals develop severe post-encephalitis neurological sequelae or succumb to the infection [4]. Since various zoonotic viruses have neurotropic potential, understanding the pathophysiology of viral encephalitis is a global priority [4, 5]. Of key interest are virus sensing mechanisms in the CNS and how these processes regulate local antiviral responses. Many emerging viruses with neurotropic potential have RNA genomes. Although multiple sensing mechanisms exist for such pathogens, retinoic acid-inducible gene-I-like receptors (RLRs) are key sensors of infection with RNA viruses [6–9]. The RLRs, including RIG-I and MDA5, signal through the single essential adaptor protein mitochondrial antiviral signaling protein (MAVS). Earlier studies showed that upon peripheral infection, RLR signaling regulates T-cell function on multiple levels, but the functional role of this signaling pathway in CNS-resident cells remains unknown [7, 9–11]. Interestingly, antigen-specific T cells isolated from brain of West Nile virus (WNV) infected *Mavs*-deficient mice showed less vigorous responses to low dosed antigen than T cells isolated from brain of WT mice [9]. These experiments suggest that MAVS signaling either selectively promotes the recruitment of T cells with an enhanced capacity to respond to low abundant antigens, or that MAVS signaling of some CNS-resident cell type(s) regulates the function of the infiltrating cells within the infected CNS.

Upon neurotropic virus infection of epithelial cells in the nasal cavity, the virus infects olfactory sensory neurons and migrates along the axons to the olfactory bulb (OB) [1, 12]. There, non-productively infected astrocytes [13, 14] mount type I interferon (IFN) responses [14, 15], which are essential to restrict virus dissemination and to promote survival [15–19]. Moreover, IFN responses of astrocytes and neurons are needed for protection [17, 19–22] by directly regulating microglia activation [19, 23]. Microglia are recruited to sites of infection by sensing nucleotides that are released from infected neurons through a P2RY12-dependent mechanism [24]. We recently showed that upon in vitro exposure of microglia to vesicular stomatitis virus (VSV), MAVS signaling is critically required to achieve full microglia activation and to mediate profound antiviral effects [25]. In *Mavs*-deficient mice, intranasal VSV instillation aused more severe disease than in WT mice and virus dissemination beyond the olfactory bulb was noticed that was correlated with the recruitment of highly inflammatory non-microglia myeloid cells into the olfactory bulb [25]. However, it remained unclear which CNS- resident cell type(s) are responsible for the enhanced susceptibility to neurotropic infection in *Mavs*-deficient mice. Following T-cell recruitment, microglia cross-present antigens to infiltrated antigen-specific CD8^+^ T cells and regulate adaptive immune responses within the infected CNS, thus limiting neuronal cell death, which mostly have low or no regenerative potential [26]. Depletion of microglia renders mice more sensitive to infection with several viruses that then show unrestricted viral spread throughout the CNS [19, 27–30]. One recent study showed that microglia depletion impairs local restimulation of infiltrated CD8^+^ T cells, which leads to reduced activity of local T cells and uncontrolled virus propagation [30]. However, CSF1R inhibition-based microglia depletion strategies have additional effects in the periphery, which makes the interpretation of results addressing T-cell responses within the infected CNS difficult [30, 31]. This aspect is relevant because brain infiltrating CD8^+^ T cells are critically required to protect against viral encephalitis [13, 26, 32–34].

In this study, we further dissected the function of MAVS in antiviral responses within the CNS by analyzing conditional mice with *Mavs*-deletion selectively either in neurons, astrocytes or long-lived CX3CR1^+^ myeloid cells such as microglia and border-associated macrophages (BAM), including perivascular and meningeal macrophages. We found that MAVS signaling of brain-resident myeloid cells is essential for locally relicensing brain-infiltrating CD8^+^ T cells and controlling VSV propagation in the brain.

## Results

### MAVS signaling of long-lived CX3CR1^+^ myeloid cells is critically required to control VSV propagation within the CNS

**Fig. 1:**
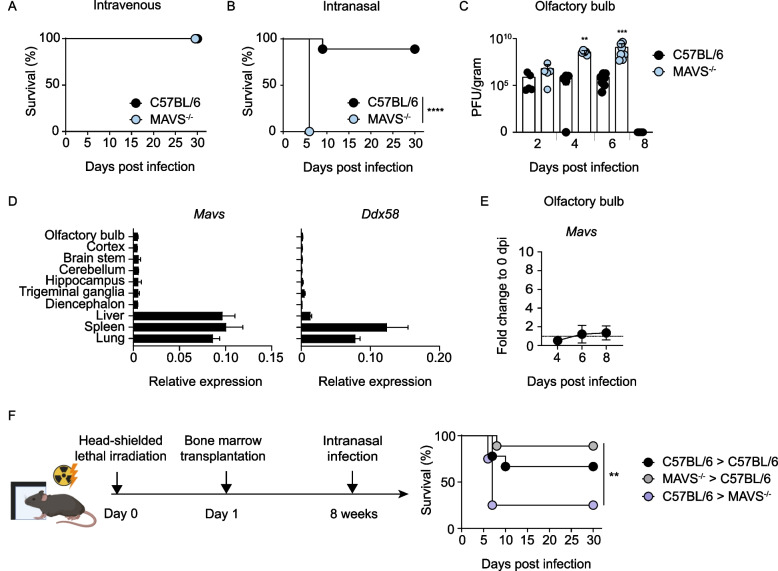
MAVS signaling is essential for protection against intranasal VSV infection. C57BL/6 and *Mavs*^*−/−*^ mice were (**A**) i.v. and (**B**) i.n. infected with 10^3^ PFU of VSV and survival was monitored for 30 days (*N* = 2, *n* = 9 per genotype, combined data) Log-rank (Mantel Cox) Test. * < 0.05, ** < 0.01, ***P < 0.001, ****P < 0.0001. **C** Virus titers were determined by plaque assay from homogenates of the OB prepared at the indicated days after infection (*N* = 2, *n* = 5–7 per genotype, combined data). Two-tailed Mann–Whitney test * < 0.05, ** < 0.01, ***P < 0.001, ****P < 0.0001. **D** *Mavs* and *Ddx58* (RIG-I) expression was determined by RT-qPCR in the indicated tissues from untreated C57BL/6 animals (*N* = 2, *n* ≥ 5, combined data). **E** *Mavs* expression was determined by RT-qPCR in OB samples from C57BL/6 mice 4, 6, and 8 days after i.n. infection (*N* = 2, *n* = 6). **F** Head-shielded mice were lethally irradiated with 10 Gy and the next day were i.v. reconstituted with the 1 × 10^7^ bone marrow cells of the indicating genotype. Following 8 weeks of recovery, mice were intranasally instilled with 10^3^ PFU of VSV and survival was monitored for 30 days (*N* = number of experiments, *n* = number of biological replicates, *N* = 2, *n* = 4–9 per genotype, combined data) Log-rank (Mantel Cox) Test. * < 0.05, ** < 0.01, ***P < 0.001, ****P < 0.0001

Previously we observed that following systemic VSV infection, the lack of either RLR or TLR signaling is fully compensated for by other PRRs [35], whereas *Mavs*^*−/−*^ mice were not able to control virus infection following i.n. VSV instillation [25, 36]. Here, we verified that WT and *Mavs*^*−/−*^ mice survived intravenous VSV infection without signs of disease (Fig. 1A), but upon i.n. infection *Mavs*^*−/−*^ mice succumbed to the infection within 6 days and most WT mice survived (Fig. 1B). Infectious virus particles could be reisolated from the OB of both WT and *Mavs*^*−/−*^ mice at day 2, 4, and 6 post i.n. VSV infection (Fig. 1C). *Mavs*^*−/−*^ mice showed steadily increasing virus titers in the OB over time and reached termination criteria by 6 days post infection (dpi), while in WT mice virus propagation was controlled in the OB, which resulted in absence of the virus by 8 dpi within the OB (Fig. 1C). In these experiments, infectious virus could not be recovered from peripheral tissues neither of WT nor *Mavs*^*−/−*^ mice including spleen, liver, and lung, except for *Mavs*^*−/−*^ mice at 2 dpi, of which some animals showed low VSV titers in the spleen that were resolved by 4 dpi (Extended Data Fig. 1A). These results confirmed that systemic VSV infection is controlled in a MAVS-independent manner, but upon i.n. VSV infection MAVS is required to control CNS infection.

Considering the indispensable role of MAVS for the control of CNS infection with VSV, we profiled *Mavs* gene expression in peripheral tissues and in different brain regions. Under homeostatic conditions, low basal expression levels of *Mavs* and *Ddx58* (RIG-I) were detected in various regions of the CNS, whereas higher expression levels were found in peripheral tissues (Fig. 1D). Also, in the VSV-infected OB, *Mavs* expression levels stayed low (Fig. 1E). Thus, despite overall low *Mavs* expression in the CNS, MAVS signaling is of key relevance to constrain virus replication and to restrict dissemination in the CNS upon i.n. VSV infection. During viral encephalitis, a broad range of immune cells infiltrate the infected CNS. To assign MAVS function to brain-resident or infiltrating cells, we generated bone marrow chimeric mice by shielding the head during the irradiation procedure, which prevents engraftment of bone marrow-derived myeloid cells within the CNS. WT mice reconstituted with WT or *Mavs*^*−/−*^ bone marrow survived i.n. VSV infection and cleared the virus similarly efficiently as observed in non-irradiated WT mice. In contrast, *Mavs*^*−/−*^ mice that were reconstituted with WT bone marrow cells showed increased sensitivity to i.n. infection (Fig. 1F). Overall, these data indicate that MAVS signaling of immune cells infiltrating the infected CNS is not needed for protection against viral encephalitis, but MAVS signaling of brain-resident cells is critical.

**Fig. 2:**
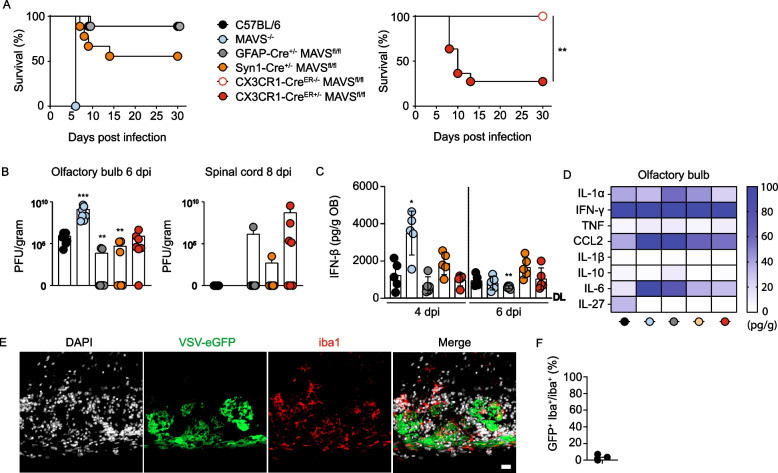
MAVS signaling of microglia is required to control VSV encephalitis. C57BL/6 (WT), *Mavs*^*−/−*^, GFAP-Cre^±^MAVS^fl/fl^, Syn1-Cre^±^MAVS^fl/fl^, CX3CR1-Cre^ER−/−^MAVS^fl/fl^ mice and CX3CR1-Cre^ER±^MAVS^fl/fl^ mice were i.n. VSV infected with 10^3^ PFU (**A-D**).** A** Survival of infected mice was monitored for 30 days (*N* = 2, *n* = 9-11 per genotype, combined data). Log-rank (Mantel Cox) Test. * < 0.05, ** < 0.01, ***P < 0.001, ****P < 0.0001. The color code shown in the legend for single genotypes of mice applies to A-D. **B** The OB and spinal cord of infected mice were prepared 6 and 8 days post infection, respectively, and viral load was determined from lysates by a plaque assay (*N* = 2, *n* ≥ 5 per genotype, combined data). The dataset of day 6 post infection OB plaques for C57BL/6 and *Mavs*^*−/−*^ mice is the same as shown in Fig. 1C. **C** The olfactory bulb of infected mice was prepared 4 and 6 dpi and IFN-β was determined from lysates by an ELISA method (*N* = 2, *n* ≥ 5 per genotype, combined data). **B – C** Two-tailed Mann–Whitney test * < 0.05, ** < 0.01, ***P < 0.001, ****P < 0.0001. **D** The OB of infected mice was prepared 6 days post infection and a flow cytometry-based bead cytokine array was performed from the lysates. The heat map shows the concentration of the indicated cytokines as the mean value from mice of different genotypes (*N* = 2, *n* = 5 per genotype, combined data). **E** WT mice were intranasally infected with 10^3^ PFU of VSV-eGFP and 6 days later histological analysis was performed from the olfactory bulb, VSV-eGFP (green) and iba1^+^ (red) within the glomerular cell layer of the OB (representative data from *n* = 3). **F** Percentage of GFP^+^ iba^+^ on total iba^+^ cells from (E) (*n* = 3)

To identify CNS-resident cell subsets that require *Mavs* expression for the control of VSV infection, we generated conditional mice with a selective *Mavs* ablation either in neurons (Syn1-Cre^±^ MAVS^fl/fl^), astrocytes (GFAP-Cre^±^ MAVS^fl/fl^), or long-lived CX3CR1^+^ myeloid cells such as microglia and BAMs (CX3CR1-Cre^ER±^ MAVS^fl/fl^ mice that were tamoxifen-treated, kept for 8 weeks and then were further analyzed). Upon i.n. VSV instillation, WT and GFAP-Cre^±^ MAVS^fl/fl^ mice survived the infection, while Syn1-Cre^±^ MAVS^fl/fl^ mice showed moderately and CX3CR1-Cre^ER±^ MAVS^fl/fl^ mice massively enhanced susceptibility to lethal VSV infection when compared with Cre negative littermates (Fig. 2A and Extended Data Fig. 2A). These results indicated that MAVS signaling of long-lived CX3CR1^+^ myeloid cells such as microglia and BAMs, and to a lesser extent of neurons, was needed to control VSV infection in the CNS. Viral loads within the OB were enhanced in infected *Mavs*^*−/−*^ mice on 6 dpi, whereas in infected GFAP-Cre^±^ MAVS^fl/fl^, Syn1-Cre^±^ MAVS^fl/fl^, and CX3CR1-Cre^ER±^ MAVS^fl/fl^ mice viral loads were similarly elevated or even lower than in WT mice (Fig. 2B). Furthermore, virus titers were detected in the spinal cord of several CX3CR1-Cre^ER±^ MAVS^fl/fl^ mice on 8 dpi, which is the time point when such mice start to develop severe disease as indicated by hind limb paralysis and other signs of sickness (Fig. 2B). Surprisingly, higher viral loads did not correlate with defective IFN-β responses. In fact, mice of all genotypes tested mounted notable IFN-β responses on 4 dpi, while *Mavs*^*−/−*^ mice showed enhanced IFN-β responses in the OB [15]. On 6 dpi, IFN-β responses within the OB were decreased when compared with 4 dpi. Notably, on 6 dpi only infected GFAP-Cre^±^ MAVS^fl/fl^ mice showed significantly lower IFN-β responses within the OB than WT controls (Fig. 2C). In accordance with a previous study [15], IFN-α responses remained undetectable within the OB after i.n. VSV infection in all transgenic mouse lines tested (Extended Data Fig. 2B). Analysis of other pro-inflammatory cytokines revealed overall similar protein levels in the OB of the analyzed mouse strains (Fig. 2D). To address whether myeloid cells were productively virus infected following i.n. VSV instillation and whether this could explain the enhanced sensitivity of CX3CR1-Cre^ER±^ MAVS^fl/fl^ mice, WT mice were infected with a recombinant eGFP-expressing VSV strain (VSV-eGFP) and the OB was analyzed by immunohistology. The majority of iba1^+^ myeloid cells within the glomerular cell layer of the OB were not eGFP-positive, indicating that myeloid cells are not a primary target of VSV upon i.n. VSV infection (Fig. 2E and F). Moreover, the virus tropism in *Mavs*^*−/−*^ mice was similar as in WT mice (Extended Data Fig. 2C) suggesting that cell type selective MAVS deficiency would also not affect the viral tropism. Thus, the enhanced susceptibility of CX3CR1-Cre^ER±^ MAVS^fl/fl^ mice was not associated directly with virus replication and sensing in long-lived CX3CR1^+^ myeloid cells such as microglia and BAMs, but it was rather mediated by indirect effects.

### Antigen cross-presentation by microglia requires MAVS signaling during viral encephalitis

**Fig. 3:**
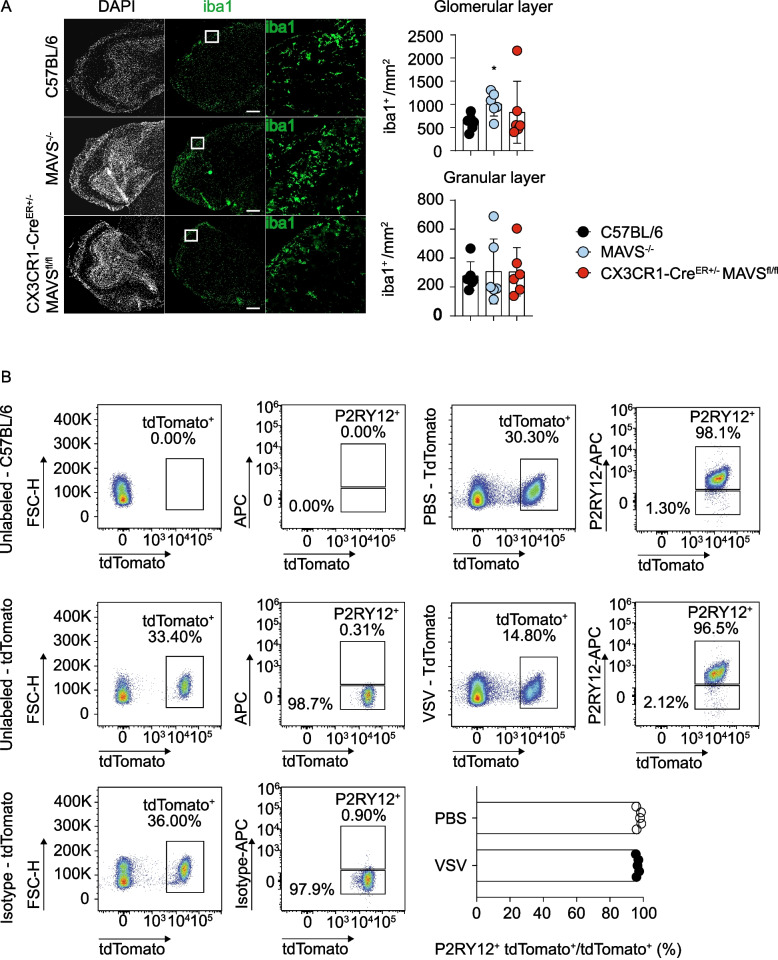
Normal myeloid cell density in mice with microglia-selective MAVS deletion.** A** Representative histology and quantification of distribution of iba1^+^ cells within the OB C57BL/6 (WT), *Mavs*^*−/−*^, and CX3CR1-Cre^ER±^MAVS^fl/fl^ mice intranasally infected with 10^3^ PFU of VSV at 6 days post infection in the glomerular and the granular cell layer (*N* = 2, *n* ≥ 6 per genotype). Two-tailed Mann–Whitney test * < 0.05, ** < 0.01, ***P < 0.001, ****P < 0.0001. **B** CX3CR1-Cre^ER±^tdTomato^wt/st^ were i.n. instilled with PBS or with 10^3^ PFU of VSV, brains were extracted at 6 days post treatment and brain-resident immune cells were isolated and immunolabelled. Percentage of P2RY12^+^ tdTomato^+^ counts on total tdTomato^+^ cells in PBS and VSV treated mice. (*N* = 2, *n* = 5 per condition, representative data)

**Fig. 4:**
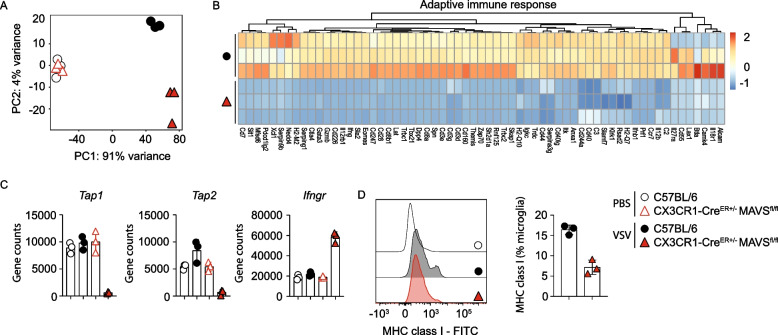
Selective MAVS deletion in microglia impairs the expression of genes relevant for cross-presentation. C57BL/6 (WT) and CX3CR1-Cre^ER±^MAVS^fl/fl^ mice were intranasally instilled with PBS or 10^3^ PFU of VSV and on 6 days post treatment the olfactory bulb was prepared, immune cells were isolated and CD45^+^CD11b^low^Ly6C^−^P2RY12^+^ microglia were directly sorted into 350 µl of β-mercaptoethanol-containing RA1 buffer for RNA extraction and subsequent bulk sequencing. **A** Principal component analysis of RNA-seq data from uninfected (PBS) and infected microglia from C57BL/6 (WT) and CX3CR1-Cre^ER±^ MAVS^fl/fl^ mice. Each dot represents data from a single mouse.** B** Heat map of relative expression of differentially expressed genes that are relevant in adaptive immune response pathway with a log_2_-fold change cut-off between microglia from C57BL/6 (WT) and CX3CR1-Cre^ER±^ MAVS^fl/fl^ mice treated with PBS or 10^3^ PFU of VSV. **C** Gene counts of *Tap1, Tap2, and Ifngr* gene from CD45^+^CD11b^low^Ly6C^−^P2RY12^+^ cells from C57BL/6 (WT) and CX3CR1-Cre^ER±^ MAVS^fl/fl^ mice infected with 10^3^ PFU of VSV. **D **Flow cytometry histogram plots and percentage of MHC class I expression in total microglia counts within the OB of C57BL/6 (WT) and CX3CR1-Cre^ER±^MAVS^fl/fl^ mice i.n. instilled with 10^3^ PFU of VSV at 6 dpi (*n* = 3 per genotype)

Flow cytometric analysis of CNS-resident immune cells in the OB at 6 dpi, which is the peak of the immune response, revealed similar abundance of leukocytes and myeloid cells, including infiltrating monocytes, dendritic cells, B cells, CD4^+^ and CD8^+^ T cells and resident microglia, in WT and CX3CR1-Cre^ER±^ MAVS^fl/fl^ mice (Extended Data Fig. 3). Immunohistological analysis of iba1^+^ cells in the OB of mice with CX3CR1-selective *Mavs* deficiency in long-lived brain-resident myeloid cells indicated that the overall myeloid cell density was similar in the glomerular and granular cell layer of the OB as in infected WT mice. Interestingly, infected *Mavs*^*−/−*^ mice showed even enhanced counts of iba1^+^ cells at 6 dpi, which is the time point when these mice reached termination criteria (Fig. 3A). To confirm that P2RY12^+^ microglia are targeted by tamoxifen induced CX3CR1-Cre^ER^ recombination, we performed fate mapping experiments with CX3CR1-Cre^ER±^ tdTomato^wt/st^ mice. P2RY12^+^ microglia were predominantly positive for tdTomato after PBS treatment and VSV infection indicating that the tamoxifen inducible CX3CR1-Cre^ER^ microglia targeting approach robustly labels P2RY12^+^ microglia (Fig. 3B). Since *Mavs* deficiency of long-lived CNS-resident CX3CR1^+^ myeloid cells such as microglia and BAMs is essential for protection and did not affect myeloid cell density, we next analyzed the transcriptome of microglia, which were known to be the more abundant population within the long-lived CX3CR1^+^ brain macrophages that were targeted in CX3CR1-Cre^ER±^ MAVS^fl/fl^ mice [37]. For this purpose, we sorted CD45^+^CD11b^low^Ly6C^−^P2RY12^+^ microglia from the OB of mock-treated and i.n. VSV infected mice at 6 dpi in order to exclude P2RY12^−^ perivascular macrophages from the subsequent analysis (Extended Data Fig. 4) [38]. Principal component analysis revealed that *Mavs* deficiency did not affect the transcriptomic profile of microglia under homeostatic conditions (Fig. 4A). In contrast, the transcriptomic profiles of microglia isolated from infected WT and CX3CR1-Cre^ER±^ MAVS^fl/fl^ mice clearly differed from each other (Fig. 4A and Extended Data Fig. 4A). Analysis of the top 10 differentially regulated pathways revealed enrichment of pathways related to chromatin and nucleosome organization in microglia derived from WT infected mice [39], which further highlighted the activated status of microglia during infection (Extended Data Fig. 4B). In contrast, microglia derived from CX3CR1-Cre^ER±^ MAVS^fl/fl^ mice showed de-enriched pathways related to T-cell activation and adaptive immune responses, implying that MAVS deficiency of microglia might affect T-cell responses. In depth analysis of the genes implicated in adaptive immune response pathways revealed a complete shut-off of genes that are involved in cross-presentation such as *Tap1, Tap2,* while *Ifngr* that is known to promote cross-presentation was highly enriched only in MAVS deficient microglia (Fig. 4B and C). Since downregulation of TAP transporters may affect the overall surface expression levels of MHC class I, we performed flow cytometry of immune cell suspensions from the OB of VSV infected control and CX3CR1-Cre^ER±^ MAVS^fl/fl^ mice on 6 dpi. Upon intranasal VSV infection CX3CR1-Cre^ER±^ MAVS^fl/fl^ mice showed reduced MHC class I expression on microglia when compared with WT controls (Fig. 4D). Overall, our data indicate that long-lived CX3CR1-selective *Mavs* deficiency does not affect myeloid cell proliferation and accumulation, but regulates the transcriptomic profile of microglia regarding expression of genes that are relevant for cross-presentation and the overall surface expression levels of MHC class I molecules. Thus, MAVS signaling potentially affects the microglia function of cross-presenting antigen to antigen-specific T cells, which recently was proposed to be a hallmark of microglia during viral encephalitis [26].

### The transcriptome of CD8^+^ T cells is affected by Mavs deficiency of long-lived CX3CR1^+^ myeloid cells during VSV infection

**Fig. 5:**
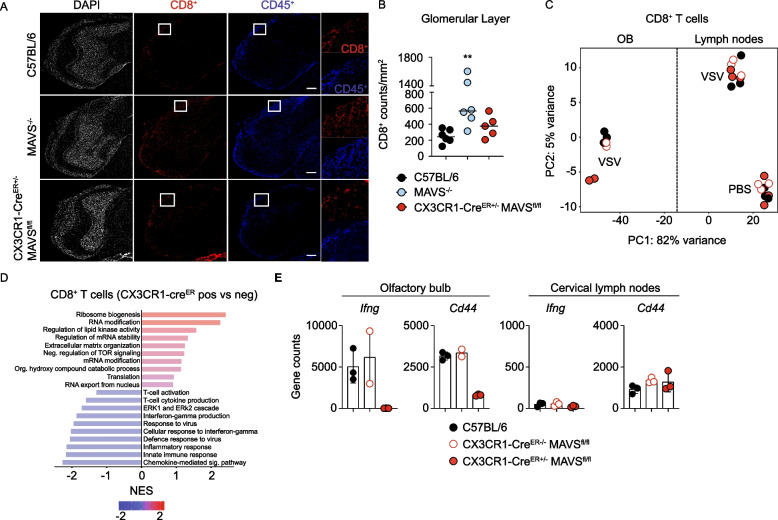
MAVS signaling of long-lived CX3CR1+ myeloid cells defines the transcriptomic profile of CNS infiltrating CD8^+^ T cells during viral encephalitis. C57BL/6 (WT), *Mavs*^*−/−*^ and CX3CR1-Cre^ER±^ MAVS^fl/fl^ mice were intranasally infected with 10^3^ PFU of VSV and mice were prepared 6 days post infection. **A** Histological analysis of CD45^+^ and CD8α^+^ within the OB (representative data, *N* = 2, *n* ≥ 5 per genotype). Small insets were selected and magnified for better visualization from the glomerular layer of the OB where we observe the vast majority of immune infiltrating CD45^+^ and CD8a^+^ cells. **B** Quantification of CD8^+^ T cells within the glomerular layer of the OB from the experiment in Fig. 5A. (*N* = 2, *n* ≥ 5 per genotype, combined data). Two-tailed Mann–Whitney test * < 0.05, ** < 0.01, ***P < 0.001, ****P < 0.0001. **C—E** C57BL/6 (WT), CX3CR1-Cre^ER−/−^MAVS^fl/fl^ and CX3CR1-Cre^ER±^MAVS^fl/fl^ mice were intranasally instilled with PBS or 10^3^ PFU of VSV and on 6 days post treatment the OB and the cervical lymph nodes were prepared, immune cells were isolated and CD8β^+^ T cells were directly sorted into 350 µl of β-mercaptoethanol RA1 buffer for RNA extraction and subsequent bulk sequencing. **C** Principal component analysis of CD8^+^ T cells from the OB and the cervical lymph nodes of C57BL/6 (WT), CX3CR1-Cre^ER−/−^MAVS^fl/fl^ and CX3CR1-Cre^ER±^MAVS^fl/fl^ mice i.n. infected with 10^3^ PFU of VSV or with PBS instilled. Each dot represents a single mouse. **D** Comparative pathway analysis of top 10 differentially enriched pathways of CD8^+^ T cells from CX3CR1-Cre^ER−/−^MAVS^fl/fl^ and CX3CR1-Cre^ER±^MAVS^fl/fl^ mice i.n. infected with 10^3^ PFU of VSV. **E** Gene counts of *Ifng and Cd44* gene from CD8^+^ T cells from C57BL/6 (WT), CX3CR1-Cre^ER−/−^MAVS^fl/fl^ and CX3CR1-Cre^ER±^MAVS^fl/fl^ mice infected with 10^3^ PFU of VSV. Each dot represents a single mouse

To further analyze whether selective *Mavs* deficiency in long-lived CX3CR1^+^ myeloid cells affects T-cell responses within the CNS, we examined recruitment of CD8^+^ T cells to the infected CNS as a hallmark of protection against lethal CNS infection [13, 26, 32, 33, 40]. Analysis of CD45^+^ and CD8^+^ cells in the OB from infected WT, *Mavs*^*−/−*^ and CX3CR1-Cre^ER±^ MAVS^fl/fl^ mice revealed that *Mavs*^*−/−*^ mice showed significantly more CD8^+^ T cells within the OB than WT mice, whereas CX3CR1-Cre^ER±^ MAVS^fl/fl^ mice showed only moderately increased numbers of CD8^+^ T cells within the OB (Fig. 5A, B). These data indicated that long-lived CX3CR1-selective *Mavs* deficiency did not affect the recruitment of CD8^+^ T cells to the infected CNS, which was in accordance with our earlier observation that leukocytes required neuronal MyD88-dependent chemokines for recruitment to the infected CNS [13]. Correspondingly, these results raised the question of whether CD8^+^ T cells, which were recruited to the infected CNS, were fully functional. To answer this, we sorted CD8^+^ T cells from the cervical lymph nodes and the OB of infected WT, CX3CR1-Cre^ER−/−^ MAVS^fl/fl^ and CX3CR1-Cre^ER±^ MAVS^fl/fl^ mice as well as CD8^+^ T cells from the cervical lymph nodes of PBS treated mice and performed RNA sequencing (RNA-seq) analysis. Principal component analysis revealed that CD8^+^ T cells residing in the draining lymph nodes segregated from the CD8^+^ T cells that infiltrate the infected CNS parenchyma, which indicated a fulminant transcriptional shift during the CNS infiltration process. Of note, CD8^+^ T cells in the OB sorted from Cre-positive littermates segregated from the CD8^+^ T cells of WT and Cre-negative littermates showing that *Mavs* deficiency of long-lived CX3CR1^+^ cells affects the T cell transcriptome (Fig. 5C). Pathway analysis revealed that CD8^+^ T cells from infected CX3CR1-Cre^ER±^ MAVS^fl/fl^ mice showed de-enriched pathways of T-cell activation and signaling when compared with negative littermates (Fig. 5D, E). Selective gene counts of transcripts relevant for CD8^+^ T-cell function such as *Ifng* and *Cd44* indicated that long-lived CX3CR1-selective *Mavs* deficiency was associated with significantly diminished transcription of these genes when compared with CD8^+^ T cells from infected WT and Cre-negative littermates (Fig. 5E). Interestingly, these genes were not affected in peripheral CD8^+^ T cells from the draining lymph nodes of CX3CR1-Cre^ER±^ MAVS^fl/fl^ mice (Fig. 5E). Thus, the above transcriptomic studies highlight the significance of MAVS signaling specifically in long-lived CX3CR1^+^ myeloid cells within the infected CNS to locally license infiltrated CD8^+^ T cells.

## Discussion

Although the CNS is well shielded by various barriers, many viruses evolved strategies to enter the CNS and cause viral encephalitis [41]. Virus infections in the CNS must be controlled by well calibrated responses that cause as little CNS pathology as possible and that are still suitable to restrict virus propagation. Our study supports the concept that brain-infiltrating T cells are relicensed locally by long-lived CX3CR1^+^ myeloid cells in a MAVS-dependent manner. We provide evidence that cross-presentation by P2RY12^+^ microglia is regulated by cell autonomous MAVS signaling. Further characterization of CD8^+^ T cells with aberrant transcriptional profiles detected within the OB of i.n. VSV instilled mice devoid of *Mavs* selectively in long-lived CX3CR1^+^ myeloid cells indicated that CD8^+^ T cells were not properly restimulated. However, in this study we did not further dissect whether also BAMs contributed to this phenotype since here we only focused on the transcriptomic analysis of P2RY12^+^ cells from the OB. By analyzing sorted P2RY12^+^ cells we excluded the possibility of perivascular macrophage contamination, whereas only a very minor proportion of dural macrophages may be P2RY12^+^ in the OB [38, 42]. Therefore, there is the theoretical possibility that BAMs may also have a role in cross-presentation, as suggested by others [31]. Interestingly, peripheral infection with neurotropic viruses induces responses that redundantly involve Toll-like receptor (TLR) and RLR signaling, and which can compensate for each other [35]. Our recent data together with this study clearly indicate that TLR and RLR signaling within the infected CNS have distinct and non-redundant functions that do not compensate for each other. Such differences in the TLR and RLR dependence of responses in the CNS and the periphery can be explained by the distinct viral tropisms in the CNS and in the periphery [13, 43].

Our data highlight the non-redundant significance of signaling during CNS infection, whereas after systemic infection *Mavs* deficiency was compensated by other PRRs [35]. In accordance with previous observations [44], we found higher gene expression levels of RLR components in peripheral tissues than in the CNS. This observation would need further analysis during CNS homeostasis and disease, since MAVS signaling is involved in several cellular functions besides virus sensing and can be directly affected by metabolic changes [45, 46]. The finding that IFN-I responses within the OB are not significantly affected by MAVS deletion in long-lived CX3CR1^+^ cells together with the observation that CX3CR1-Cre^ER±^ MAVS^fl/fl^ mice succumb to the infection at the time point when T-cell responses are needed to restrict virus propagation imply that cell–cell communication between microglia and infiltrating T cells is critically relevant. Correspondingly, and as we did not expect to detect changes in IFN-I responses in mice with selective MAVS deficiency in brain cells, we did not further analyze IFN-I responses in the serum of such mice. Earlier experiments by others with WNV-infected bone marrow chimeric mice indicated that MAVS-mediated protection is conferred by the radio-sensitive compartment [9]. In that study, whole body irradiation of mice was performed, that is known not only to eliminate peripheral immune cells but also to reduce the density of microglia in the brain [47], while at later time points Ly6C^hi^CCR2^+^ infiltrating monocytes engraft the CNS parenchyma due to the availability of the niche [48]. Of note, such brain-engrafting bone marrow-derived myeloid cells show distinct transcriptomic profiles and reveal differential responsiveness to stimuli when compared with microglia [49]. We want to highlight, that in this study we focused on the analysis of the impact of selective *Mavs* deletion in long-lived CX3CR1^+^ myeloid cells on transcriptional traits of CNS-infiltrating T cells, but we did not study T cell-selective *Mavs* deletion. Importantly, several other studies highlighted that RLR signaling on the T cell level is required to exhibit full T cell functionality [7, 9–11, 50]. In accordance with our experiments, CSF1R-mediated microglia depletion experiments during neuroinvasive WNV infection resulted in diminished local restimulation of CD8^+^ T cells within the brain and enhanced susceptibility to infection [19, 27–30]. These observations suggest that microglia may regulate the function of CD8^+^ T cells within the infected brain. Unfortunately, such models are not ideal to study the role of CD8^+^ T-cell priming and function due to impaired myeloid cell homeostasis under conditions of peripheral immune cell depletion [31]. To that extent our genetic ablation of MAVS in long-lived CX3CR1^+^ CNS-resident cells such as microglia and BAMs did not affect the transcriptomic traits of CD8^+^ T cells in the periphery suggesting that upon i.n. VSV infection MAVS signaling of CNS myeloid cells is responsible for altered transcriptomic profiles of brain infiltrating T cells. Importantly, upon MAVS deletion we did not detect impaired infiltration with CD8^+^ T cells, further supporting the concept that MyD88-dependent chemokine responses drive T-cell infiltration during viral encephalitis [13, 51]. By analyzing sorted P2RY12^+^ cells we associated the effects observed in the CD8^+^ T cells with microglia. It is also not known whether deficiency of MAVS signaling of long-lived CX3CR1^+^ CNS cells might affect the outcome of T-cell responses due to potential differences in the pool of presented epitopes that would drive cross-presentation from BAMs in our infection model [31]. In a model of WNV-induced neurocognitive dysfunction driven by CD8^+^ T cell mediated synaptic elimination, long-lived CX3CR1^+^ CNS cells mediate the phenotype through IFN-γ receptor (IFNGR)-signaling. Deletion of *Ifngr* selectively in long-lived CX3CR1^+^ CNS cells ameliorated the cognitive decline suggesting an interaction between long-lived CX3CR1^+^ CNS cells such as microglia, and CD8^+^ T cells in an IFN-II dependent manner [52]. Consistent with these earlier findings, our results show that *Mavs*-deficient microglia display marked upregulation of IFNGR when compared with WT microglia. Notably, in WNV infection experiments, antigen-specific CD8^+^ T cells showed diminished in vitro functional activity if sorted from brain of *Mavs*^*−/−*^ animals compared with T cells sorted from brain of WT animals [9]. These results are in accordance with our observations and further highlight that CD8^+^ T cells maintain their function upon interaction with MAVS competent brain-resident cells. To that extent, reduced MHC class I surface expression in MAVS deficient microglia suggests that indeed the transcriptomic signatures observed in brain infiltrating CD8^+^ T cells may arise from the diminished microglia cross-presentation capacity. Interestingly, we did not observe reduced myeloid cell density in the OB of mice with a selective MAVS deficiency in long-lived brain-resident myeloid cells. This suggested that despite downregulation of MHC class I molecules on microglia no subsequent NK-cell mediated myeloid cell depletion was triggered, probably due to incomplete MHC class I downmodulation [53]. In future studies it will be interesting to address whether the pool of presented peptides differs in MAVS deficient and competent microglia under conditions of infection and inflammation. Moreover, targeting RLR signaling with the RIG-I agonist stem loop RNA 14 (SLR14) inhibited tumor growth in murine cancer models [54, 55], which was associated with enhanced anti-tumor CD8^+^ T cell responses. Of note, under such conditions, SLR14 was mainly taken up by myeloid cells suggesting that triggering of MAVS signaling in antigen presenting cells affected the transcriptomic and functional responses of CD8^+^ T cells potentially by a similar mechanism as during VSV infection of the CNS. Recently, it was discovered that RLR-related inborn errors of immunity predispose patients to develop viral encephalitis [56, 57]. The case of *GTF3A* mutations that control the transcription of the host-derived pseudogene *RNA5SP141*, which is known to bind to RIG-I, is of particular interest [57]. However, the role of host-derived pseudogenes in microglia biology is still unclear. Importantly, triggering of RLR signaling in microglia upon i.n. VSV instillation is not associated with productive or abortive microglia infection suggesting that RLR signaling is either triggered during the phagocytosis of infected material or by yet unsolved endogenous mechanisms that are in place during macrophage activation [26].

Taken together, our results highlight MAVS-dependent induction of the antigen presentation machinery of long-lived CX3CR1^+^ CNS cells such microglia as a novel critical mechanism of microglia-CD8^+^ T cell interaction during viral encephalitis. This observation may pave the way for targeted manipulation of the MAVS signaling platform to modulate adaptive immune responses within the CNS compartment.

## Materials and methods

### Mice

*C57BL/6JOlaHsd* were used as WT control mice and B6.STOCK-Mavs^(tm1Tsc)^ [58] as *Mavs*^*−/−*^ mice. B6.Cg-Tg(Syn1-Cre)^Par^ [59], B6N.B6CBA-Tg(GFAP-Cre-IRES-LacZ)^DG^ [60] and B6.Cg-Cx3Cr1^tm2.1(cre/ERT2)Jung^ [61, 62] were intercrossed with B6J.MAVS^tm1Rm1^ (MAVS^fl/fl^) [63] in order to generate Cre expressing mice that are homozygous for the floxed allele. B6.Cg-Cx3Cr1^tm2.1(cre/ERT2)Jung^ were intercrossed with Gt(ROSA)26Sor^tm9(CAG−tdtomato)HZE/J^ [64] in order to generate heterozygous reporter mice. All mice were screened for absence of germline *Mavs* deletion by PCR. To this end the set of 5´-CTTCCTTCACCCTTGGACCTTCT-3′ and 5´- TGACTGGGTGTAGACTCTGTACT-3′ primers was used for the detection of the floxed allele and the set of 5´-ATACTGTTAAAGCACAGGGCTGG-3′ and 5´- TGACTGGGTGTAGACTCTGTACT −3′ primers was used for the detection of *Mavs* germline deletion. For generation of mice with a microglia-selective *Mavs* deletion, the animals were subcutaneously injected with tamoxifen at the age of six weeks. Tamoxifen (Sigma-Aldrich) was diluted in corn oil (Sigma-Aldrich) to a final concentration of 20 µg/µl and 2 injections of 200 µl were performed with an interval of 48 h. After tamoxifen administration the animals were left untreated for 8 weeks. All mouse strains used in this study were bred under specific pathogen-free conditions in the central mouse facility of the Helmholtz Centre for Infection Research, Brunswick, and at TWINCORE, Centre for Experimental and Clinical Infection Research, Hannover, Germany. Mouse experimental work was carried out using mice older than 8 weeks in compliance with regulations of the German animal welfare law (Tierschutzgesetz – TierSchG) of Lower Saxony State Office for Consumer Protection and Food Safety (Niedersächsisches Landesamt für Verbraucherschutz und Lebensmittelsicherheit) with protocol numbers 14/1594, 18/2899, and 19/3292.

### Infection

Mice were anaesthetized by intraperitoneal injection of 1.6% ketamine (WDT eG) and 0.08% xylazine (cp-pharma) in physiological saline (0.1667 mg/g ketamine and 0.00834 mg/g xylazine, 100 µL/10 g mouse body weight intraperitoneally). Mice were intranasally instilled with a total volume of 10 μl of 10^3^ PFU of VSV Indiana or VSV-eGFP in sterile PBS in both nostrils. For intravenous VSV infection, mice were warmed up within their cages with a red-light lamp for 1–2 min and then they were placed in a restrainer. Mice were intravenously injected with 100 μl of 10^3^ PFU of VSV Indiana in sterile PBS. VSV Indiana (Mudd-Summers isolate) was originally obtained from D. Kolakofsky (University of Geneva, Switzerland), while VSV-eGFP was kindly provided by Gert Zimmer (University of Bern, Switzerland) [65].

### Perfusion

Mice were anaesthetized by intraperitoneal injection of 1.6% ketamine (WDT eG) and 0.08% xylazin (cp-pharma) in physiological saline (0.3334 mg/g ketamine and 0.01668 mg/g xylazin, 200 µl/10 g mouse body weight intraperitoneally). Vital signs such as breathing rate and heartbeat decline and the cessation of the toe reflex were closely examined before the initiation of the perfusion protocol. When reflex signs were no longer detectable, mice were fixated horizontally to a preparation block. The thorax was opened and an incision was performed within the diaphragm. Subsequently, a butterfly cannula was inserted within the lower part of the left ventricle of the heart and a slight incision was made in the right ventricle to promote blood release from the circulation. Thereafter, 10 ml of cold PBS were flushed through the vasculature and for subsequent histological analysis of tissues an additional 10 ml of cold 4% PFA (Carl Roth) was flushed in without the application of a fixed flow rate and pressure.

### Determination of viral loads

Organs were extracted from perfused mice and stored in lysing matrix tubes type A or D (MP Biomedicals) at −80 °C in MEM supplemented with 5% FCS and 1% Glutamax (Gibco LifeTechnologies). The samples were thawed and homogenized in a FastPrep24 homogenizer (MP Biomedicals) for 30 s (4 m/s). The organ homogenates were inoculated in a tenfold serial dilution onto Vero cell monolayers (Vero B4 from DSMZ) in 24 well plates that were seeded with Vero cells in a density of 200,000 cells/well 24 h prior to the inoculation. After inoculation, samples were incubated for 1 h at 37 °C. To block floating infectious viral particles, the monolayers were overlaid with 1% Methylcellulose (VWR International) for 24 h at 37 °C. Finally, the overlay was aspirated and the monolayers were stained and fixed with a crystal violet solution (Merck) for 2 h.

### Irradiation and generation of bone marrow chimeric mice

Mice were head-shielded and were lethally irradiated with 10 Gy and the following day, they were i.v. reconstituted with 1 × 10^7^ bone marrow cells of the indicated genotype. Irradiated mice were infected after 8 weeks of recovery.

### Histology

Mice were transcardially perfused with 10 ml of cold PBS and 10 ml of cold 4% PFA. Brains were dissected and individually incubated in 4% PFA for 2 h at room temperature (RT). Brains were then transferred to 30% sucrose buffer overnight in a rotator at 4 °C. Subsequently, brains were fully immersed into OCT (Sakura) and were frozen on top of dry ice. Sagittal cryosections of 7 μm were cut using the cryotome and placed in cryoslides stored at −20 °C until immunolabeling. Initially, cryosections were hydrated with 0.05% Triton X 100 in PBS for 15 min at RT. Blocking was performed with 5% Donkey Serum (Sigma-Aldrich) and 0.05% Triton X 100 (Sigma-Aldrich) in PBS for 1 h at RT. Primary antibodies were incubated with 0.05% Triton X 100 in PBS overnight at 4 °C. The following antibodies were used: goat anti-mouse iba1 (1:500, Abcam), rabbit anti-mouse iba1 (1:500, Wako), mouse anti-mouse CD8a-PE (1:100, Biolegend), mouse anti-mouse CD45-APC-Cy7 (1:100, Biolegend). Secondary antibodies were incubated with 0.05% Triton X 100 in PBS for 1 h at RT and nuclei were counterstained with DAPI (Invitrogen) in PBS for 2 min at RT. The secondary antibodies used were the following: donkey anti-goat 568 (1:1000, Invitrogen), donkey anti-rabbit 568 (1:1000, Invitrogen), donkey anti-goat 488 (1:1000, Invitrogen). In between, double washing steps were performed with 0.05% Triton X 100 in PBS. Finally, cryosections were mounted with one drop of DAKO mounting buffer (DAKO). Slides were visualized with the Zeiss AxioScan.Z1 and the Olympus FV-1000 microscopes. Original images were analyzed and quantified with the ZENLite software and adjustment of brightness, contrast, color balance and post processing were performed with the Fiji software.

### Isolation of immune cells from the CNS and the olfactory bulb

For the complete brain immune cell isolation mice were transcardially perfused with 10 ml of cold PBS and each individual brain was isolated and transferred to GentleMACS C type tubes (Miltenyi) together with 2 ml RPMI medium with 5% FCS, 5 μl rDNAse (Macherey–Nagel) and 10 μl collagenase type D (Invitrogen). The C tubes were placed in the GentleMACS Dissociator (Miltenyi) and were run in the three brain specific programs with 8 min incubation time at 37 °C in between the programs. Upon enzyme digestion, samples were washed with 1 × PBS and centrifuged at 300 rcf for 6 min. Pellets were resuspended in a 70% Percoll gradient (Sigma-Aldrich) and were underlaid with a 37% and 30% Percoll gradient sequentially and were centrifuged for 30 min at 500 × g without brakes. The immune cells were isolated from the layer between the 70% and 37% Percoll gradient with a 1 ml pipette and the cell suspension was transferred into a new 15 ml falcon tube and washed with MACS buffer (Miltenyi). Therefore, the cell suspension was centrifuged again at 300 rcf for 6 min and the supernatant was aspirated leaving only 500 μl in the tube to resuspend the immune cells.

For the isolation of immune cells from the olfactory bulb (OB), mice were transcardially perfused with 10 ml of cold PBS and the OB was prepared. Upon transfer into a pre-cooled glass homogenizer potter with 3 ml of 1.5% HEPES in HBSS medium (Gibco LifeTechnologies), the OB tissue was homogenized for approximately 30 s. The homogenate was flushed through a 70 μm cell strainer into a 50 ml falcon tube. The strainer was further flushed with 2 ml of 1.5% HEPES in HBSS medium and samples were centrifuged at 400 rcf for 10 min at 4 °C. Supernatants were discarded and the pellet was resuspended in a 37% Percoll gradient and transferred in a new 15 ml falcon tube. Samples were centrifuged at 150 rcf for 30 min at 4 °C without brakes. Supernatants were discarded and cells were resuspended in 500 μl of PBS and then transferred in FACS tubes for further analysis. Data were analyzed with FlowJo v10.4.

### Flow cytometry

Upon immune cell isolation samples were incubated with 1 µl of anti-CD16/CD32 (rat mAb 2.4G2, mouse Fc block BD) for 10 min at 4 °C. Then they were immunolabelled with either 50 μl of the antibody mix or isotype mix for 20 min at 4 °C. Afterwards, 50 μl of AccuCheck Counting-Beads (ThermoFischer Scientific) were added and washed with 1 ml of FACS buffer. Cells were centrifuged for 6 min at 300 rcf and supernatants were removed. Finally, cells were resuspended in 50 μl of 1% PFA. UltraComp eBeads™ Plus Compensation Beads (BD Biosciences) were used for single labeling compensation controls. The SP6800 cytometer and ID7000 Spectral Analyzers (both Sony) were used to acquire the samples for flow cytometry. Data were analyzed and processed with FlowJo software (BD Biosciences). The following antibodies were used: Pacific Blue CD45.2 (clone 104, 1:100, Biolegend), BV510 CD11b (clone M1/70, 1:50, Biolegend), APC P2RY12 (clone S16007D, 1:25 Biolegend), FITC MHC class I (clone M1/42, 1:50, Biolegend), PerCP-Cy5.5 anti-mouse CD8b (clone YTS156.7.7, 1:50 Biolegend), BV421 CD11c (clone N418, 1:50 Biolegend), BV570 CD4 (clone RM4-5, 1:50 Biolegend), PE-Dazzle 594 anti-mouse Ly6C (clone HK1.4, 1:50 Biolegend), PE-Cy5 B220 (clone RA3-6B2, 1:50 Biolegend). Data were analyzed with FlowJo v10.4.

### Immune cell fluorescence sorting

Upon isolation of immune cells, samples were incubated with 1 µl of anti-CD16/CD32 (rat mAb 2.4G2, mouse Fc block BD) for 10 min at 4 °C and then were immunolabelled with either 50 μl of the antibody mix or isotype mix for 20 min at 4 °C. The cells were washed with 1 ml of FACS buffer, centrifuged for 6 min at 300 rcf, and the supernatant was removed. Finally, cells were resuspended in 100 μl of FACS buffer. Cells were sorted directly into 350 μl of RA1 buffer (Macherey–Nagel) with 1% β-mercaptoethanol (Merck) using the FACSAria™ Fusion Cell Sorter (BD Biosciences). The following antibodies were used: Pacific Blue CD45.2 (clone 104, 1:100, Biolegend), APC-Cy7 CD11b (clone M1/70, 1:50, Biolegend), APC P2RY12 (clone S16007D, 1:25 Biolegend), PE anti-mouse CD8b (clone YTS156.7.7, 1:50 Biolegend), PE-Cy7 CD3 (clone 145-2c11, 1:50 Biolegend), AF700 anti-mouse Ly6C (clone HK1.4, 1:50 Biolegend).

### Cytokine array

Organs were extracted from perfused mice and stored in lysing matrix tubes type D containing 500 µl MEM supplemented with 5% FCS and 1% Glutamax at −80 °C for at least 24 h before the array. Samples were thawed and homogenized in a FastPrep24 homogenizer for 30 s (4 m/s). Homogenates were used to determine the cytokine concentration with the LEGENDplex Mouse Inflammation Panel kit (Biolegend, cat. 740,446), following the manufacturer’s protocol. Pre-dilution steps were not performed in these assays. Quantification of cytokine concentrations was analyzed with the LSRII flow cytometer and data were evaluated with LEGENDplex V8.0 software from Biolegend.

### IFN-β and IFN-α determination by ELISA

Mouse Verikine IFN-β ELISA kit (PBL, cat. 42,400) was used for quantifying IFN-β in supernatants of murine organ homogenates. IFN-α was determined by Mouse Verikine IFN-α ELISA kit (PBL, cat. 42,120) in organ homogenates from mice. All of the kits were used according to the manufacturer’s protocol.

### RNA extraction

Organs were isolated from mice and were collected into Lysing Matrix A or D tubes containing 350 μl of RA1 buffer with 1% β-mercaptoethanol for OB tissues. Samples were stored at −80 °C. Organs were homogenized for 60 s with a speed of (4 m/s) in the FastPrep24 homogenizer and the NucleoSpin® RNA Kit (Macherey–Nagel) was used to extract the RNA according to the manufacturer’s instructions. Genomic DNA was digested with reconstituted rDNAse for 15 min according to the manufacturer´s instructions (Macherey–Nagel). RNA was eluted in 20 μl of nuclease-free water and RNA concentration and quality was measured on NanoDrop 1000 Spectrophotometer (ThermoFischer Scientific). For the cDNA synthesis, PrimeScript™ RT Master Mix kit (Takara) was used according to the manufacturer’s instructions. The reaction mixture was prepared in order to obtain a concentration of 10 ng/μl RNA template together with 2 μl PrimeScript™ RT and RNAse –free water (Macherey–Nagel) for a final volume of 10 μl. The samples were incubated for 15 min at 37 °C and then heated for 10 s at 87 °C. The SensiFAST SYBR® No-ROX Kit (Bioline) was used to perform RT-qPCR. All of the samples were run in triplicates and the specificity of the amplification was controlled by inspecting the melting curves. Furthermore, reactions with either just H_2_O or the master mix instead of samples were run to ensure that there was no contamination in the used reagents. For each run a final volume of 20 μl reaction mixture (10 μl SensiFAST SYBR®, 0.8 μl 200 μM Forward primer, 0.8 μl 200 μM Reverse primer, 6.4 μl H_2_O and 2 μl of 1.25 ng/ml template) was pipetted into the plate. The following primers were used (*Mavs* forward 5’-CTGGCTGATCAAGTGACTCG-3’, *Mavs* reverse 5’-AATGCAGAGGGTCCAGAAAC-3’, *Gapdh* forward 5’-GTGGCAAAGTGGAGATTGTT-3’ *Gapdh* reverse 5’-CTTGACTGTGCCGTTGAATT-3’). The plate was centrifuged for 10 s and the LightCycler480 (Roche) was used to perform the qRT-PCR.

### Sequencing analysis

RNA from tissues specified at each experiment figure legend was extracted as described above. RNA integrity was assessed using RNA 6000 pico Kit Bioanalyzer (Agilent Technologies) and cDNA libraries were constructed using NEBNext Low input RNA Library Prep Kit for Illumina (NEB, E6420) following manufacturer’s instructions at the sequencing facility of the Helmholtz Centre for Infection Research. The extracted RNA was sequenced on an Illumina NovaSeq-6000 platform with a 50 bp paired-end read configuration at the sequencing facility of the Helmholtz Centre for Infection Research. Quality of raw fastq-files was assessed using FastQC software (version 0.11.9) and mapped to reference genome assembly of *Mus musculus* (Mm10) from Ensembl using STAR v2.5.4b software. Only reads with unique mapping were considered for downstream analysis. Gene abundance of mapped reads was calculated using Mm10 mouse gene annotation from Ensembl with Feature Counts v1.6.0 software. Differential expression analysis was performed using DESeq2 package in R software (version 1.26.0). Following a fitting with negative binomial generalized linear model (GLM), the Wald test was used to test significance of gene expression differences as a function of samples at an absolute log_2_-fold change threshold of 2. To control false discovery rate (FDR), the Wald test *p*-values were adjusted to multiple comparisons using the Benjamini–Hochberg procedure. To display expression levels of selected gene signatures between samples, raw counts were normalized based on median of ratios method in DESeq2. Differentially expressed genes represented as heatmaps were generated using standardized function of normalized counts from DESeq2 analysis. Functional annotation of differentially expressed gene signatures was performed in Gene Ontology, subcategory “Biological process”.

### Statistics

Cell numbers, viral loads and cytokine concentrations were compared by the two tailed Mann–Whitney U test. For survival analysis, the Mantel–Cox survival analysis with log-rank statistics was used. A *P* value of 0.05 was considered statistically significant. For statistical analysis, the software package GraphPad Prism Version 9.0 was used. Data are shown as mean ± SD unless stated otherwise in the figure legend.

## Supplementary Information

Supplementary Material 1: Extended Data Fig. 1. MAVS signaling is not required to control VSV dissemination to peripheral organs upon intranasal virus instillation. C57BL/6 and *Mavs*^*−/−*^ mice were i.n. VSV infected with 10^3^ PFU of VSV and liver, spleen, and lung was prepared at the indicated days. (A) Virus titers were determined by plaque assay in liver, spleen, and lung homogenates (*N* = 2, *n* ≥ 5 per genotype, combined data). (B) Virus titers were determined by plaque assay in olfactory bulb (OB), cerebrum (CR), brain stem (BS), cerebellum (CRBL), liver (LI), and spleen (SPL) homogenates dissected at the end of the experiment (30 dpi) or at the time point of exclusion criteria from one of the two survival experiments (Fig. 1F). Two-tailed Mann–Whitney test * < 0.05, ** < 0.01, ***P < 0.001, ****P < 0.0001. Extended Data Fig. 2. MAVS signaling within the infected CNS is essential for viral restriction and host protection. (A) Virus titers were determined by plaque assay in olfactory bulb (OB), cerebrum (CR), brain stem (BS), cerebellum (CRBL), liver (LI), spleen (SPL) and lung (LU) homogenates dissected at the time point of exclusion criteria from the survival experiment (Fig. 2A) from i.n. VSV infected MAVS^−/−^, Syn1-Cre^±^ MAVS^fl/fl^ and CX3CR1-Cre^ER±^ MAVS^fl/fl^. (B) The OB of infected mice was prepared 4 days post infection and IFN-α was determined from lysates by an ELISA method (*N* = 2, *n* ≥ 5 per genotype, combined data). (C) Primary data in dot plot graphs per cytokine as shown in the heat map of Fig. 2D. (D)* Mavs*^*−/−*^ mice were intranasally infected with 10^3^ PFU of VSV-eGFP and 6 days later histological analysis was performed from the olfactory bulb, VSV-eGFP (green) and P2RY12^+^ (red) within the glomerular and granular cell layer of the OB (representative data from *n* = 2). Extended Data Fig. 3. *Mavs* deficiency in microglia does not affect leukocyte recruitment into the infected OB. (A) Flow cytometry density plots (B) and quantification of immune cell subsets within the OB of C57BL/6 (WT) and CX3CR1-Cre^ER±^ MAVS^fl/fl^ mice i.n. instilled with 10^3^ PFU of VSV at 6 dpi (*n* = 3 per genotype). Extended Data Fig. 4. Depletion of *Mavs* in microglia affects the microglia transcriptomic profile during infection. (A) Heat map of relative expression of differentially expressed genes with a log_2_-fold change cut-off between microglia from C57BL/6 (WT) and CX3CR1-Cre^ER±^MAVS^fl/fl^ mice i.n. infected with 10^3^ PFU of VSV. (B) Comparative pathway analysis of the top 10 differentially enriched pathways of microglia from C57BL/6 (WT) and CX3CR1-Cre^ER±^ MAVS^fl/fl^ mice infected with 10^3^ PFU of VSV.

## Data Availability

The RNA-seq data is available under the Gene Expression Omnibus (GEO) accession numbers GSE301540 and GSE301690. The authors confirm that the data underlying the findings are fully available without restriction upon request to the lead contact.
