# Supplementary figures and images for: MAVS signaling of long-lived brain-resident myeloid cells is needed during viral encephalitis to adjust the transcriptome of CNS infiltrating CD8+ T cells
Source: J Neuroinflammation. 2025 Jul 7;22:175. doi: 10.1186/s12974-025-03497-1 (PMC12232705; doi:10.1186/s12974-025-03497-1)

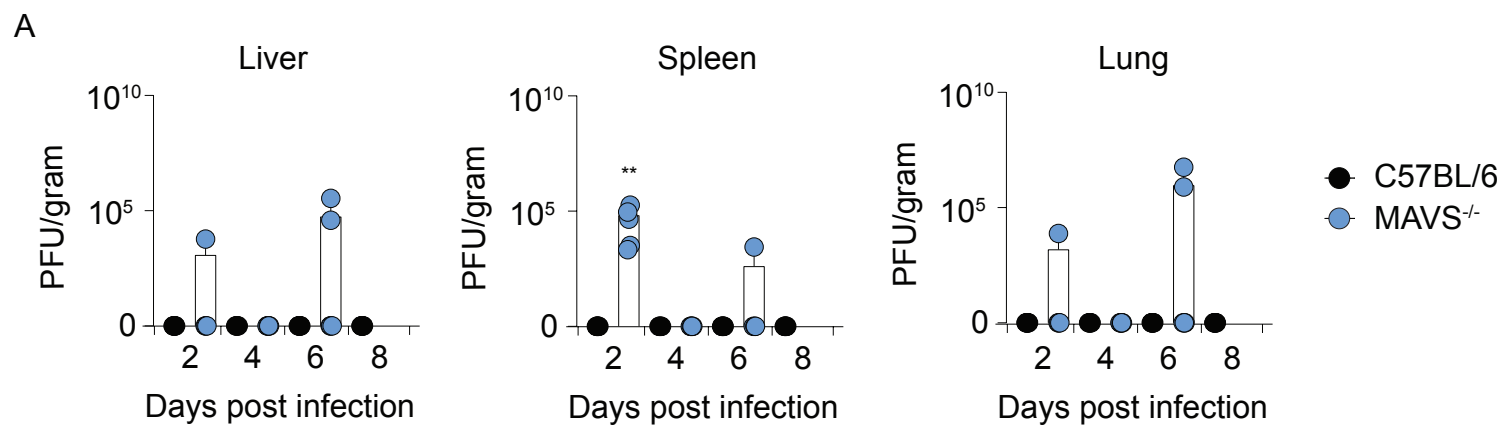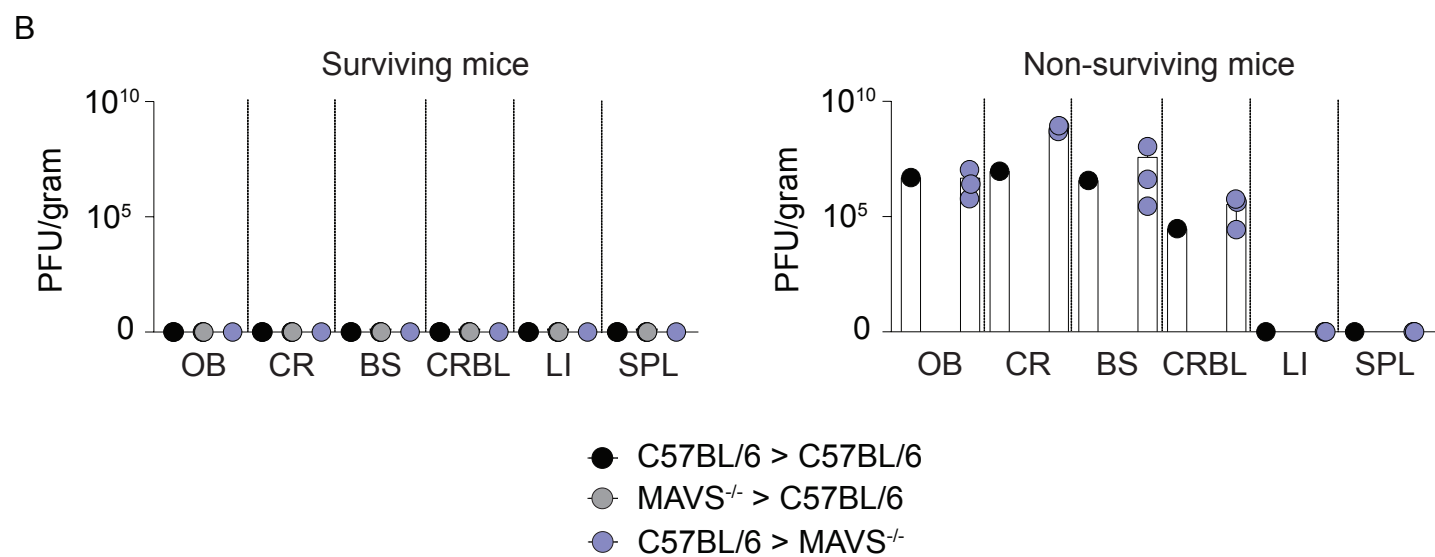

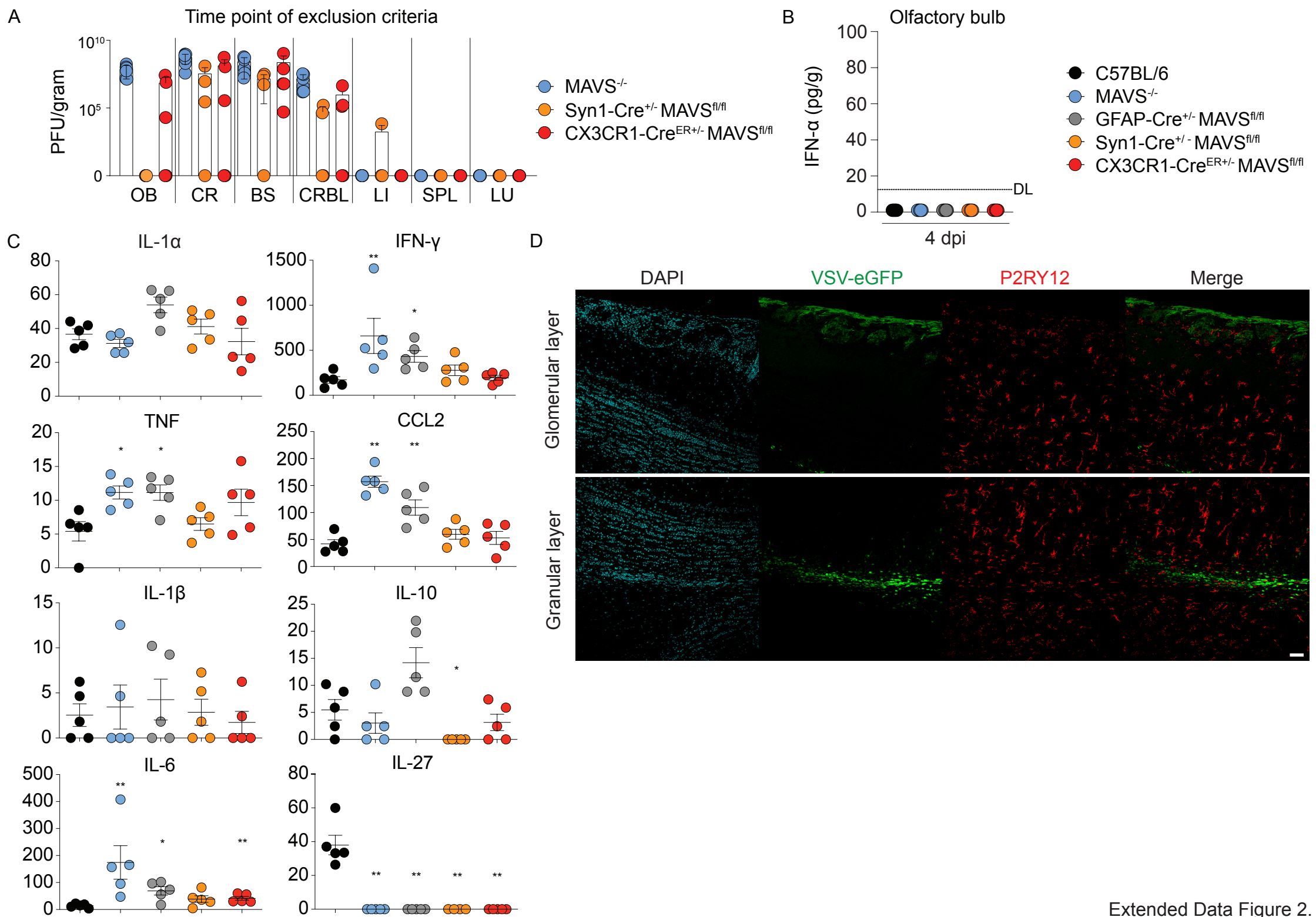

Extended Data Figure 2.

A

VSV infected

C57BL/6

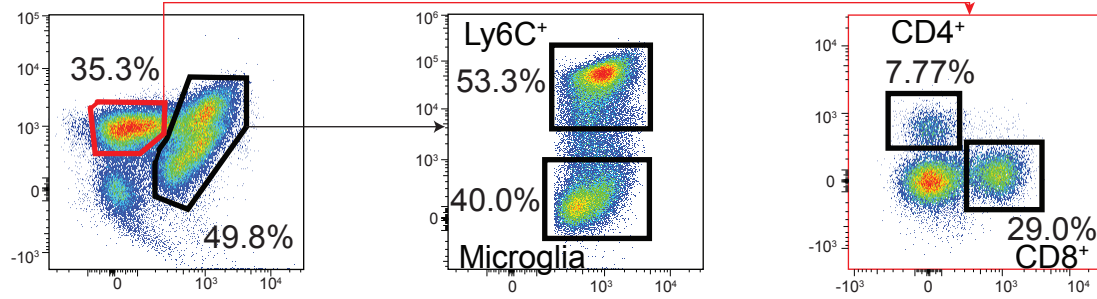CX3CR1-Cre<sup>ER+/-</sup>  
MAVS<sup>fl/fl</sup>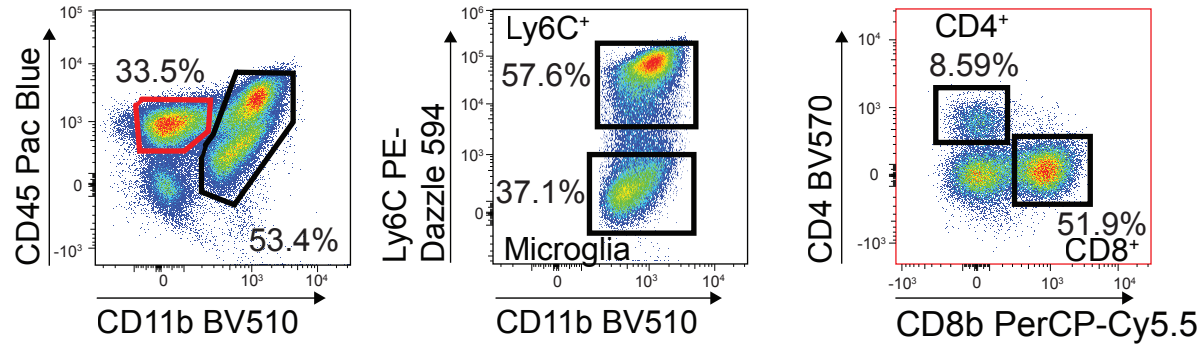

B

Olfactory bulb

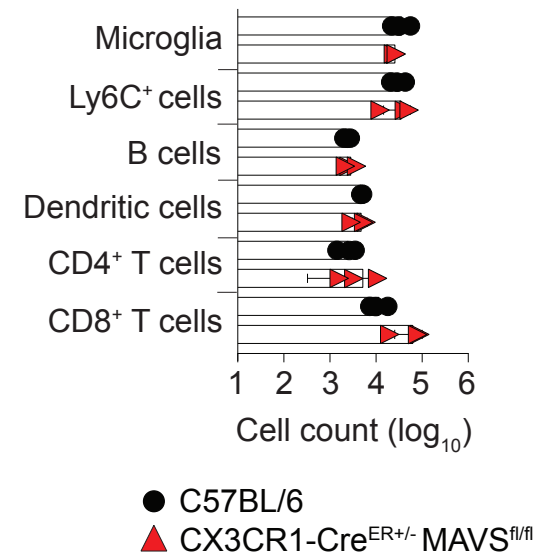

A

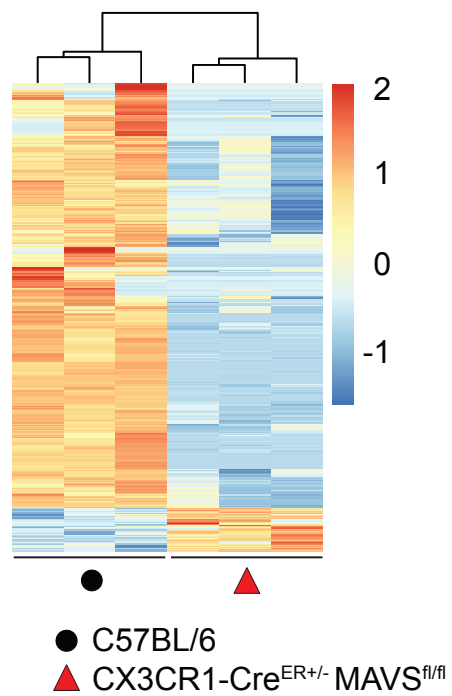

B

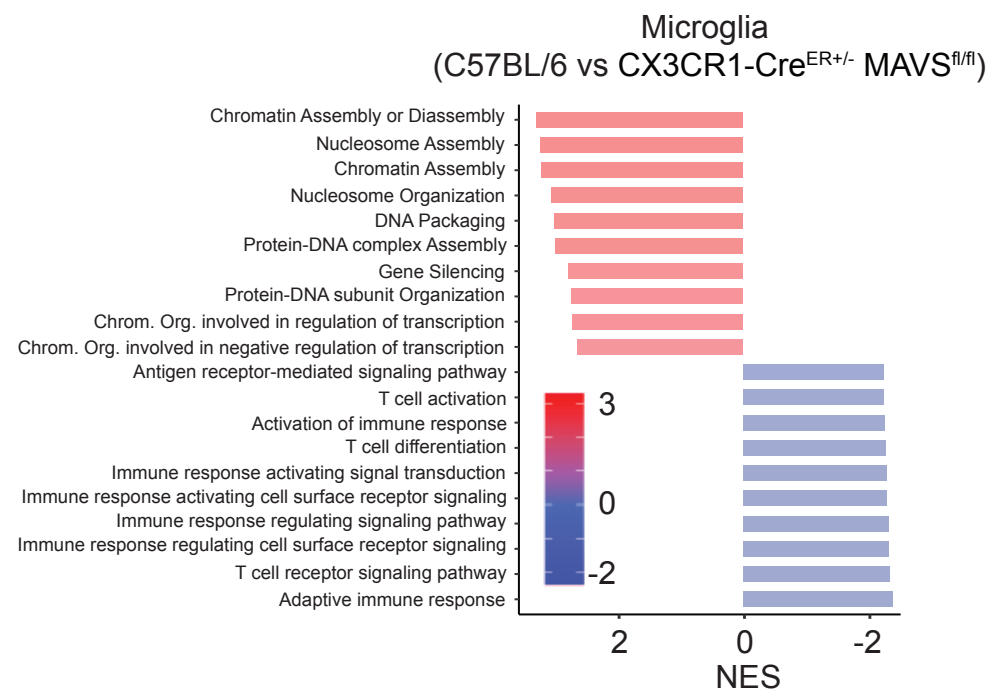

Supplement: Supplementary file 1 — Supplementary Material 1: Extended Data Fig. 1. MAVS signaling is not required to control VSV dissemination to peripheral organs upon intranasal virus instillation. C57BL/6 and Mavs−/− mice were i.n. VSV infected with 103 PFU of VSV and liver, spleen, and lung was prepared at the indicated days. (A) Virus titers were determined by plaque assay in liver, spleen, and lung homogenates (N = 2, n ≥ 5 per genotype, combined data). (B) Virus titers were determined by plaque assay in olfactory bulb (OB), cerebrum (CR), brain stem (BS), cerebellum (CRBL), liver (LI), and spleen (SPL) homogenates dissected at the end of the experiment (30 dpi) or at the time point of exclusion criteria from one of the two survival experiments (Fig. 1F). Two-tailed Mann–Whitney test * < 0.05, ** < 0.01, ***P < 0.001, ****P < 0.0001. Extended Data Fig. 2. MAVS signaling within the infected CNS is essential for viral restriction and host protection. (A) Virus titers were determined by plaque assay in olfactory bulb (OB), cerebrum (CR), brain stem (BS), cerebellum (CRBL), liver (LI), spleen (SPL) and lung (LU) homogenates dissected at the time point of exclusion criteria from the survival experiment (Fig. 2A) from i.n. VSV infected MAVS−/−, Syn1-Cre± MAVSfl/fl and CX3CR1-CreER± MAVSfl/fl. (B) The OB of infected mice was prepared 4 days post infection and IFN-α was determined from lysates by an ELISA method (N = 2, n ≥ 5 per genotype, combined data). (C) Primary data in dot plot graphs per cytokine as shown in the heat map of Fig. 2D. (D) Mavs−/− mice were intranasally infected with 103 PFU of VSV-eGFP and 6 days later histological analysis was performed from the olfactory bulb, VSV-eGFP (green) and P2RY12+ (red) within the glomerular and granular cell layer of the OB (representative data from n = 2). Extended Data Fig. 3. Mavs deficiency in microglia does not affect leukocyte recruitment into the infected OB. (A) Flow cytometry density plots (B) and quantification of immune cell subsets within th [file 12974_2025_3497_MOESM1_ESM.pdf]
